# Anatomical Researches on Pulmonary Emphysema

**Published:** 1839-04

**Authors:** 


### Art. XIV.

*Recherches Anatomiques sur l'Emphysème Pulmonaire.* Par le Dr. Lombard, Médecin de l'Hôpital Civil et Militaire de Genève.

*Anatomical Researches on Pulmonary Emphysema.* By Dr. Lombard, &c. &c.—*Geneva*, 1837. 4to. pp. 20.

The chief object of the author of this memoir is to elucidate the anatomical history of pulmonary emphysema, in order to give greater precision to the opinions of those who have previously investigated this part of the disease. Dr. Lombard begins by dividing pulmonary emphysema into three forms,—the *vesicular*, *lobular*, and *lobar*, excluding the interlobular; because, existing external to the "pulmonary tissue," he does not consider it "as a lesion of the organs of respiration, and, consequently, that it ought not to be included under the same denomination." Were we disposed to recommend the separation, nosologically considered, of interlobular from pulmonary emphysema, we should certainly suggest a more scientific ground of distinction than that assigned by Dr. Lombard; viz. the very different nature and mechanism of the disease in each. The three forms of pulmonary emphysema designated above by Dr. Lombard were well known to the illustrious author who first made the profession acquainted with this disease, and as accurately if not so minutely described by him and several other succeeding pathologists. The extent of the emphysema is the chief ground of the distinction of the three forms of the disease, and of their respective appellations. In the first form, the emphysema is limited to a few isolated vesicles; in the second, it affects an entire lobule, which is the most common of the three forms; and, in the third, it occupies the whole of a lobe, one or both lungs. Besides these differences in the extent of the emphysema. Dr. Lombard points out others derived from the anatomical characters of the disease; the most of which will be found in the works of Laennec, Andral, and others, as characterizing the different stages of the disease. As, however, the opinions of Dr. Lombard on the nature of pulmonary emphysema are founded on the results of his anatomical investigations, we shall briefly state the anatomical characters given by him of each form of the disease.

The essential anatomical character of the first form is a certain degree of enlargement of a number of the air-cells, the size of which is greater in reality than in appearance, from the portion contained in the substance of the lung being larger than that seen externally. When the enlargement of the air-cells is considerable, it is always owing to a communication having been formed between several contiguous ones, from destruction of their parietes. This circumstance, the destruction of the parietes of a number of the air-cells, and a lateral communication between them, constitutes the distinctive anatomical character of the second form. It is by far the most common form of the disease, and the description given of it is clear and accurate. The lobulated character which it so frequently assumes is clearly traced; and the fact noticed of the peripheral portion of the lobules being always most affected,—that is to say, the degree of the emphysema being always greatest at the surface of the lung. We do not, however, agree with the author in the explanation which he gives of it,—viz. "the greater extensibility of the pleura than of the tissues situated in the substance of the lung." We apprehend that the greater size of the air-cells at the surface of the lung, and, *a fortiori*, the production of the large pyriform and globular appendices met with in aggravated cases of the disease, is a necessary consequence of the physical law which so powerfully contributes to the accomplishment of the function of respiration,—viz. the pressure of the atmosphere, which, during inspiration and expiration, but more especially during the former, must tend more or less to carry outwards, to distend and elongate the pleura pulmonalis and contiguous air-cells. To this influence might, perhaps, be added another, that of the confined air, the elasticity of which, probably increased, operating in the peripheral direction, in which there is the least opposing force. The third form of emphysema presents two varieties: "In the first variety all the lobules are emphysematous, but in different degrees;" its anatomical characters are the same as those of the second form. In the second variety, "the whole of a lobe, or even of one lung, is transformed into a spongy-looking body, apparently in a state of hypertrophy; but the augmentation of volume does not depend on increase, but rather on atrophy of the areolar tissue, which, having lost its elasticity, has become distended by the inspired air. The size of the air-cells is variously increased, combined in some parts with rupture of their parietes, and giving rise to the formation of anfractuosities; the blood-vessels are obliterated, or at least diminished in size and number; the pulmonary tissue being pale, and almost exsanguineous; the interlobular cellular tissue has disappeared; and hence the surface of the lung presents a uniform aspect, without intersections throughout the whole of a lobe." The only fact of importance noticed in the description of the anatomical characters of this form of emphysema is the *obliteration or diminution in size and number of the blood-vessels;* and on this circumstance he founds his opinion on the origin and nature of the disease. "Direct observation," he observes, "leads us to recognize in pulmonary emphysema two anatomical circumstances essential to its existence,—1, the destruction of a considerable part of the intervesicular parietes; 2, the obliteration of almost all the capillary vessels in the emphysematous portion of the lung." The reader will no doubt feel some surprise at this conclusion of the author, as it has been already distinctly stated that the first form of pulmonary emphysema—viz. the vesicular—passes into the second, or lobular, when the enlarged cells in the former communicate with one another, from rupture of their parietes. In the first form of emphysema there is, therefore, according to our author's own statement, simply dilatation, not rupture, of the air-cells. Indeed, that pulmonary emphysema, whether existing in a portion of a lobule, a whole lobe, or in both lungs, consists simply in dilatation of the air-cells, without any other appreciable physical alteration, is a fact fully established; and it is even in the most extensive forms of the disease that this state of simple dilatation is most conspicuous. It is, however, as Dr. Lombard has justly observed, always complicated with destruction of the intervesicular parietes when carried beyond a certain extent. Both states of the air-cells are, indeed, generally met with, sometimes the one, sometimes the other morbid condition predominating; but we hold the opinion that the former condition, or dilatation of the air-cells in pulmonary emphysema, always *precedes* the destruction of their parietes—in one word, that *dilatation* of the air-cells, from whatever cause arising, with *retention* of the inspired air, constitutes the essential physical characters or conditions of pulmonary emphysema. To this state succeeds the destruction of the intervesicular parietes, and, consequently, the extension of the disease under an entirely new form; the consequence, in fact, of atrophy, as stated by our author, from obliteration of the vascular structure of the lungs. This explanation of the production of pulmonary emphysema accords with that given by Dr. Carswell, in Fasciculus x. of his work on the Elementary Forms of Disease, under the section entitled "Atrophy from a diminished supply of blood." This author has, besides, pointed out the *cause* of the morbid condition of the capillary circulation which gives rise to the atrophy,—viz. the long-continued compression of the capillary vessels by the confined air; a mechanical cause, in fact, which is well known to occasion the same modification of nutrition in various other organs. While alluding to atrophy as a powerful and frequent cause of the *extension* of pulmonary emphysema, we think it of sufficient importance to observe, that the application of the term to that state of the lungs in old people produced by atrophy is extremely improper. Our author considers this state as similar in its nature to his second form of emphysema in young persons; and previous pathologists, more especially Andral, have committed the same error. The destruction of the intervesicular structure of the lungs from the atrophy of old age, although depending on obliteration of the capillary circulation, obviously does not, from what we have stated, constitute pulmonary emphysema. There is not necessarily *dilatation* of the air-cells, and no *retention* of the inspired air in the atrophiated vesicular structure; consequently no increase in the bulk of the lung or difficulty of breathing as the necessary concomitant. On the contrary, there is not only a greater or less diminution in the bulk of the lungs, but also a corresponding one in the capacity of the thorax; changes, the various degrees of which, and the relations which they bear to each other, have been well illustrated by the researches of MM. Hourmann and Dechambre. We trust, therefore, that pathologists, at least, will no longer apply the term emphysema to that state of the lungs in old people produced by atrophy.

It may not be irrelevant to our present purpose to advert here to a state of the lungs not unfrequently, but incorrectly, described as emphysema. In this state the lungs do not collapse when the cavity of the thorax is laid open; they even sometimes project beyond the cut edges of the ribs, and they communicate, when pressed, the same sensation as when affected with emphysema. But they are not affected with emphysema; for their vesicular structure has undergone no change of capacity, as is obvious by comparing the air-cells with those of a healthy and collapsed lung. The inflated condition of the lungs is the consequence of forced inspiration, and occurs in various diseases, but more especially in those cases of bronchitis, whether succeeding to a chronic state of the disease or not, in which the bronchial secretions are retained to such a degree as to obstruct or interrupt the egress of the air, and which, consequently, accumulating more or less rapidly, produces death by asphyxia. Obstruction to the free egress of the inspired air is certainly the most essential of the causes of emphysema; but, in the state of the lungs to which we are now alluding, the fatal effects of the obstruction are too rapid to allow of dilatation of the air-cells taking place. It is, in fact, a state of the lungs which does not differ from that which may and often does occur in strangulation, sudden closure of the glottis, or compression of the trachea: or it may be produced after death, simply by insufflation. It is, in fine, a state of *inflation*, and, from the frequency of its occurrence, from its being probably the immediate cause of death, and, to distinguish it from emphysema, ought to be noted and designated as such.

Dr. Lombard very justly, we think, discards the notion of hypertrophy of the air-cells being ever associated with emphysema; and he does so on the ground of his not having met with this change of bulk in his anatomical researches. Our objections to this notion are founded on the fact that hypertrophy and dilatation of the air-cells, when they do occur, constitute a physiological and not a pathological change; being always subservient to a corresponding and supplementary increase of the function of respiration, the very reverse of what occurs in pulmonary emphysema.

In the explanation of the signs and symptoms of pulmonary emphysema, considered in relation to the anatomical lesions, nothing of any importance has been added by Dr. Lombard to our previous knowledge on this part of the subject; nor does he suggest anything in the treatment of the disease likely to be more successful than the means generally employed.

The coloured lithographs, explanatory of the author's views, are very beautiful, and characteristic of several forms of the disease.

---

